# Optimising Cardiometabolic Risk Factors in Pregnancy: A Review of Risk Prediction Models Targeting Gestational Diabetes and Hypertensive Disorders

**DOI:** 10.3390/jcdd9020055

**Published:** 2022-02-10

**Authors:** Eleanor P. Thong, Drishti P. Ghelani, Pamada Manoleehakul, Anika Yesmin, Kaylee Slater, Rachael Taylor, Clare Collins, Melinda Hutchesson, Siew S. Lim, Helena J. Teede, Cheryce L. Harrison, Lisa Moran, Joanne Enticott

**Affiliations:** 1 Monash Centre for Health Research and Implementation, School of Public Health and Preventive Medicine, Monash University, Clayton, VIC 3168, Australia; eleanor.thong@monash.edu (E.P.T.); drishti.ghelani@monash.edu (D.P.G.); siew.lim1@monash.edu (S.S.L.); helena.teede@monash.edu (H.J.T.); cheryce.harrison@monash.edu (C.L.H.); lisa.moran@monash.edu (L.M.); 2 Faculty of Medicine, Nursing and Health Sciences, Monash University, Clayton, VIC 3168, Australia; pman0013@student.monash.edu (P.M.); ayes0004@student.monash.edu (A.Y.); 3 School of Health Sciences, College of Health, Medicine and Wellbeing, and Priority Research Centre for Physical Activity and Nutrition, University of Newcastle, Callaghan, NSW 2308, Australia; kaylee.slater@uon.edu.au (K.S.); rachael.taylor@newcastle.edu.au (R.T.); clare.collins@newcastle.edu.au (C.C.); melinda.hutchesson@newcastle.edu.au (M.H.)

**Keywords:** cardiovascular, risk prediction, pregnancy, gestational diabetes, hypertensive disorders of pregnancy, preeclampsia

## Abstract

Cardiovascular disease, especially coronary heart disease and cerebrovascular disease, is a leading cause of mortality and morbidity in women globally. The development of cardiometabolic conditions in pregnancy, such as gestational diabetes mellitus and hypertensive disorders of pregnancy, portend an increased risk of future cardiovascular disease in women. Pregnancy therefore represents a unique opportunity to detect and manage risk factors, prior to the development of cardiovascular sequelae. Risk prediction models for gestational diabetes mellitus and hypertensive disorders of pregnancy can help identify at-risk women in early pregnancy, allowing timely intervention to mitigate both short- and long-term adverse outcomes. In this narrative review, we outline the shared pathophysiological pathways for gestational diabetes mellitus and hypertensive disorders of pregnancy, summarise contemporary risk prediction models and candidate predictors for these conditions, and discuss the utility of these models in clinical application.

## 1. Introduction

Cardiovascular disease (CVD), encompassing coronary heart disease, stroke and heart failure, is the leading cause of death in women worldwide [1,2]. Beyond the traditional risk factors for CVD, including diabetes mellitus and hypertension, reproductive factors such as adverse pregnancy outcomes are increasingly associated with long-term cardiovascular health [3]. Pregnancy represents a state of increased cardiovascular stress, leading to significant metabolic and haemodynamic maternal adaptations to support foetal growth. The unmasking of metabolic disorders, such as gestational diabetes mellitus (GDM) and hypertensive disorders of pregnancy (HDP), in women with pre-existing vascular dysfunction [3], has key implications for the development of future cardiometabolic disease [4].

GDM is a form of glucose intolerance occurring during pregnancy and is increasingly prevalent, affecting between 2 to 14% of pregnancies [5]. GDM confers an increased risk of perinatal complications, including foetal macrosomia, preterm labour and increased caesarean delivery rates [6,7]. A diagnosis of GDM has long-term implications for women, where the lifetime risk of progression to type 2 diabetes mellitus (T2DM) is up to seven-fold [8]. Key findings from a systematic review and meta-analysis of over 5 million women between 1950 to 2018 showed that women with a history of GDM had a two-fold higher risk of cardiovascular events postpartum, compared with women without GDM, independent of the development of T2DM [9].

HDP, including preeclampsia (PE), complicate up to 10% of all pregnancies and represent a major cause of maternal and perinatal morbidity and mortality [10,11]. Women with a history of PE have double the risk for future CVD [12], while women with hypertensive pregnancies have a two- to eight-fold greater risk of developing chronic hypertension, compared to those with normotensive pregnancies [13].

Epidemiological studies demonstrate an increased risk of HDP in women with GDM [10] and vice versa [14], suggesting shared pathophysiological pathways, via insulin resistance (IR) and obesity. IR is an established precursor for the development of T2DM and is also thought to induce hypertension via cellular, circulatory, and neurological mechanisms [14]. Obesity increases IR and confers 4-fold [15] and 10–15% [16] increased risks for GDM and preeclampsia, respectively, compared with women of normal weight. Taken together, epidemiological data reinforces IR and obesity as putative factors and a shared link between GDM and HDP.

Prediction models have been developed with the aim of providing personalised risk assessment to identify at-risk women in early pregnancy, so that timely intervention can be delivered to mitigate adverse outcomes. However, the reliability and utility of these models for the prediction of GDM and HDP are limited. There is considerable heterogeneity between studies comparing prediction models, leading to a push for greater emphasis to be placed on methodologically sound and externally validated studies [17]. Recent advancements in the risk prediction modelling landscape may help overcome these shortcomings, including the development of the Transparent Reporting of a multivariable prediction model for Individual Prognosis Or Diagnosis (TRIPOD) initiative [18], availability of big data sets for external validation studies and updating models to optimise performance [19]. The use of sophisticated approaches, such as machine learning algorithms, may complement traditional statistical modelling and assist clinical decision making [20]. On the other hand, unsupervised machine learning techniques may help to reduce the number of predictor variables to a manageable size when dealing with a very large dataset, such as genomic biomarkers [21].

This scoping review aims to provide an updated summary of contemporary risk prediction models for GDM and HDP in women with singleton pregnancies, highlight information gaps and discuss the utility of these models in clinical application. We identify shared candidate predictors for both GDM and HDP, which can help build on established models and inform the development of a novel prediction model for composite cardiometabolic complications in pregnancy.

## 2. Methods

We conducted a systematic literature search in Ovid MEDLINE and PubMed. Articles for GDM risk prediction models were searched from 2016 to June 2021, as an update to the review by Kenelly and McAuliffe [5] on risk prediction models in GDM, published in 2016. Our search strategy incorporated the following search terms: [“Validat$.mp or Predict$.ti or Rule$.mp” OR “(Model$ or Clinical$).mp” OR “(Decision$ and (Model$ or Clinical$ or Logistic models)).mp” OR “Stratification.mp or ROC Curve/or Dicrimination.mp or cstatistic.mp or Algorithm.mp or Multivariable.mp”] AND [“diabetes, gestational” OR “(gestation* adj4 diabet*).ti, ab” OR “GDM”].

In June 2018, recommendations from the International Society for the Study of Hypertension in Pregnancy (ISSHP) were established to provide a living guideline for diagnosing and managing HDP [22]. As such, for HDP risk prediction models, the literature search included articles from 2018 to June 2021, to reflect the updates in HDP classification and diagnosis. The following keywords were employed in our literature search: [“Validat$.mp or Predict$.ti or Rule$.mp” OR “(Model$ or Clinical$).mp or Logistic Models/” OR “(Prognostic and (History or Variable$ or Criteria or Score$ or Characteristic$ or Finding$ or Factor$ or model$)).mp” OR “Stratification.mp or ROC Curve/or Dicrimination.mp or cstatistic.mp or Algorithm.mp or Multivariable.mp”] AND [“hypertension, pregnancy-induced/or pre-eclampsia/” OR “(pre eclamp* or preeclamp*).tw,kw.” OR “(hypertens* adj3 (pregnan* or gestation* or maternal)).tw,kw.”].

The literature search was limited to studies carried out in humans and published in English. For each of the searches, two independent reviewers performed the screening and data extraction.

## 3. Gestational Diabetes Mellitus

Screening and diagnostic criteria for GDM varies between countries and resource settings. Biochemical testing for GDM is most commonly undertaken between 24 to 28 weeks gestation, via an oral glucose tolerance test (OGTT), in women not known to have a previous history of diabetes [23]. Growing evidence suggests GDM screening should be performed earlier in pregnancy, as excessive foetal growth is present by the time GDM is diagnosed at 28 weeks of gestation in women of normal weight, and at 20 weeks in women with overweight or obesity [24]. A recent meta-analysis reported that a high proportion (15–70%) of GDM could be detected in the first trimester. Perinatal mortality and neonatal hypoglycaemia were significantly increased amongst women with early-onset, compared to late-onset GDM [25], potentially reflecting worse outcomes in those with unrecognized disorders of glucose metabolism prior to conception. Therefore, the International Association of Diabetes and Pregnancy Study Groups (IADPSG) recommends that women at high risk of GDM can be screened in early pregnancy, at the first prenatal visit [26]. The development and use of personalised risk scores, aimed at identifying high-risk women for hyperglycaemia in early pregnancy, may allow more time for intervention to optimise pregnancy outcomes [27]. We evaluated 23 studies on risk prediction models for GDM, published from 2016 onwards.

### 3.1. Clinical Risk Factors

The earliest first trimester prediction models developed by Teede in 2011 [4] and Van Leeuwen in 2009 [5], have shown moderate-to-good overall performance in predicting GDM. Teede and colleagues [4] developed a risk prediction tool in early pregnancy, incorporating the clinical variables of maternal age, BMI, ethnicity, family history of GDM, and past history of GDM. In this model, the validation group clinical scores achieved a sensitivity of 61.3% and specificity of 71.4%, with an area under the curve of 0.703. Van Leeuwen’s model [5] incorporated similar risk variables and achieved a sensitivity of 75% and specificity of 57.8%, with an area under the curve of 0.77. Presently, the Teede and Van Leeuwen models are regarded to be the best performing models, having been externally validated across several different populations, with consistent results [28,29]. Meertens and colleagues [29] validated 12 selected risk prediction models published between 1997 to 2017, in a Dutch cohort of 5260 women. The discriminative performance of the included models ranged from 68% to 75%, with nearly all models overestimating the risk, which improved with recalibration. Schaefer and colleagues [30] devised a scoring system based on clinical risk factors including age, BMI, weight gain and family history of GDM, which had moderate discriminative performance for GDM prediction in Chinese women. Notably, this study incorporated early weight gain as a variable, which had not been considered in other models. A prediction model by White and colleagues [31] focussed on early prediction of GDM in women with obesity (BMI ≥ 30), using clinical and anthropometric variables such as age, previous GDM, family history of T2DM, systolic BP, sum of skinfold thickness, waist-to-height and neck-to-thigh ratios, with the rationale that BMI is a poor index of fat mass. This model had moderately good discriminative performance (AUC 0.71), which was enhanced with the addition of candidate biomarkers (random glucose, HbA1c, fructosamine, adiponectin, sex hormone binding globulin and triglycerides).

In the aforementioned models, risk prediction relies on a previous history of GDM as the strongest risk factor for recurrent GDM, so that this is not applicable to women in their first pregnancy. Nulliparous women appear to have a different risk profile compared to multiparous women, where the risk of adverse birth outcomes appears to be higher in the former [32]. Furthermore, it is plausible that a previous diagnosis of GDM may increase awareness of GDM risk in subsequent pregnancies and encourage behaviour modification to ameliorate this risk. So far, only a small handful of models have been tailored to assess GDM risk in nulliparous women. Donovan and colleagues [33] rigorously developed a prediction model using a large, racially and ethnically diverse cohort of over 1 million nulliparous women, which included five risk factors (race/ethnicity, age at delivery, pre-pregnancy BMI, family history of diabetes and pre-existing hypertension). This model was validated internally and externally, achieving moderate predictive performance in nulliparous women with AUCs of 0.732 and 0.710 in the internal and external cohorts, sensitivities of 70.8% and 76.7%, and correct classification of 64.3% and 57.1%, respectively. A key strength of this model was a subgroup analysis, which assessed model performance among specific racial/ethnic groups, with particularly good discriminative ability in Hispanic and African American populations.

### 3.2. Biomarkers and Biophysical Variables

Maternal multi-marker serum screening for chromosomal aneuploidy and neural tube defects is routinely offered to women in the first and early-second trimester. The use of biologically plausible serum biomarkers may improve the ability of current GDM prediction models in early pregnancy. Novel maternal aneuploidy and lipid biomarkers, such as pregnancy-associated plasma protein A (PAPP-A), unconjugated oestriol (uE3), dimeric inhibin-A (INH) and lipocalin-2, were studied in three prediction models [34,35]. Snyder et al. found that the addition of PAPP-A only and PAPP-A, uE3 and INH to maternal characteristics (ethnicity, age at delivery, pre-pregnancy BMI) in nulliparous women was found to increase model performance slightly [34]. A combined clinical and first-trimester aneuploidy and preeclampsia screening model was studied in a multiethnic Australian cohort, where the inclusion of PAPP-A, mean arterial pressure (MAP) and uterine artery pulsatility index (UtA-PI) appeared to improve screening efficacy, albeit only marginally [36]. In this cohort, ethnicity appeared to modify biomarker association with GDM. Namely, lipocalin-2 and triglycerides performed best in Caucasian and South Asian women, respectively [35]. A two-step approach combining fasting plasma glucose and high molecular weight adiponectin at 12–15 weeks gestation in women deemed to be at high risk of GDM, was shown to improve sensitivity and predictive ability of the 2011 Teede model [37,38]. Two studies found that the addition of common, low-cost candidate biomarkers (fasting/random plasma glucose, triglycerides and HbA1c) enhanced predictive performance of early GDM in women with overweight [39] or obesity [31]. The addition of HbA1c and sex hormone binding globulin (SHBG) collected between 6 to 14 weeks of pregnancy also had a significant, albeit slight improvement, to model performance [40]. In recent years, there has been growing interest regarding genetic susceptibility to GDM and the candidacy of genetic biomarkers as screening tools for GDM. DNA methylation and microRNAs have been widely studied in GDM and hold potential as diagnostic or prognostic markers [41]. One model incorporated genetic polymorphism scores for the prediction of GDM in South Asian women, with only moderate predictive performance (AUC = 0.65) [42]. However, differences in allele frequencies between ethnicities, suggest that these genetic associations may not be reproducible across different populations. While promising, there is currently insufficient evidence to recommend the use of genetic biomarkers in the prediction of GDM. Furthermore, the cost-effectiveness of these biomarkers in GDM prediction remains limited and implementation may not be feasible in low-resource settings. Nevertheless, this remains an area of growing interest as the increasing availability of genome-wide association studies (GWAS) and Mendelian randomisation may help uncover complex, polygenic disease aetiologies [43].

### 3.3. Modifiable Risk Factors

Observational data from the past decade demonstrate that lifestyle factors, before and during pregnancy, are associated with GDM risk [44]. Targeting modifiable lifestyle factors, such as smoking, diet quality and physical activity [45] in early pregnancy, could have a tangible effect on reducing GDM risk. A recent large prospective cohort study by Gao et al. [46] found that six predictors collected at the first antenatal care visit (advanced maternal age, elevated BMI, height, systolic blood pressure (SBP) and serum alanine transaminase (ALT), and family history of diabetes in first-degree relatives) and four during pregnancy modifiable risk factors (reduced physical activity, increased sitting time at home, passive smoking, and excessive weight gain), were associated with an increased risk of GDM in Chinese women (AUC of 0.712 (95% CI: 0.682 to 0.743)). Schoenaker and colleagues [47] developed a model aimed at predicting preconception GDM risk. The authors utilised prospective data from a large Australian population-based study of 6504 nulliparous women aged 18–23 years at enrolment in 1996, who reported a pregnancy during a 19-year follow-up period. The final model included eight variables based on lifestyle and health-related characteristics, including age at menarche, proposed age at future first pregnancy, ethnicity, BMI, diet score, physical activity, polycystic ovary syndrome (PCOS) and family histories of type 1 and 2 diabetes and GDM. This model showed good discriminative ability (AUC 0.79) and high sensitivity (91%) on internal validation, albeit low specificity (46%), with the potential to predict GDM in women, even before their first pregnancy. While several studies have considered the inclusion of lifestyle factors in model development, these were not found to be significant predictors of GDM [45].

### 3.4. Machine Learning Approaches

Most prediction models for GDM have been developed using logistic regression analyses. Machine learning is increasingly emphasized as a competitive alternative to regression analysis and has the potential to outperform conventional regression, possibly by its ability to capture nonlinearities and complex interactions among multiple predictive variables [48]. Two studies assessed machine learning in GDM prediction. Wu et al. developed state-of-the-art prediction models in early pregnancy for the early intervention of GDM in over 30,000 Chinese women, where a 73-variable deep neural network model achieved high discriminative power (AUC = 0.80) [49]. A clinically cost-effective 7-variable logistic regression model which was simultaneously developed, also showed effective discriminative power (AUC = 0.77), suggesting that both machine learning and logistic regression models achieved high accuracy for predicting GDM in early pregnancy. On the other hand, Ye et al. compared machine learning approaches to logistic regression in predicting GDM in Chinese women and found that machine learning methods did not outperform logistic regression in predictive ability [48].

### 3.5. Summary

Recent GDM risk prediction models identified in the literature have employed a combination of maternal characteristics and/or biochemical variables (Table 1) to improve the sensitivity and specificity of risk prediction. Most prediction models have a strong focus on maternal demographics and clinical risk factors, demonstrating moderately good discriminative performance, particularly in high-risk groups and amongst women of diverse ethnicities. These models concurred on several well-established risk factors for GDM, such as a previous diagnosis of GDM, elevated pre-pregnancy BMI, and family history of type 2 diabetes in first-degree relatives. The addition of low-cost biomarkers obtained in the first trimester (serum triglyceride, fasting plasma glucose, HbA1c and SHBG levels) to established maternal clinical risk factors (such as age, BMI and previous macrosomia) have been shown to improve the accuracy of risk prediction. On the other hand, model performance was only marginally improved with the use of novel biomarkers, such as those employed in aneuploidy screening, and maternal adipokines. Machine learning models show promise in enhancing prediction accuracy, although they have not been shown to transcend conventional logistic regression models presently.

## 4. Hypertensive Disorders in Pregnancy

There are several sub-types of HDP, including chronic hypertension, gestational hypertension (GH) and PE. Chronic hypertension is defined as hypertension (systolic blood pressure (BP) ≥140 mm/Hg and/or diastolic BP ≥90 mm/Hg) known prior to pregnancy or presenting before 20 weeks gestation. GH is characterised by hypertension arising at or after 20 weeks gestation. PE is the occurrence of gestational hypertension with accompanying proteinuria, uteroplacental dysfunction and/or other maternal organ involvement (e.g., haematological, liver or neurological complications) in previously normotensive women [22]. HDP are a leading cause of significant mortality and morbidity for both mother and foetus, especially in cases of PE and eclampsia, a neurologic complication of severe PE [50]. Worldwide, HDP are responsible for over 50,000 maternal deaths each year, accounting for approximately 12.9% of maternal deaths in developed countries and 14.0% in developing countries [51,52]. Up to 10% of pregnancies are complicated by GH or PE [10], leading to an increased risk of foetal growth restriction, preterm birth, placental abruption and maternal end-organ dysfunction. All variations of HDP pose an increased risk of both maternal and neonatal morbidity; however, women with PE or eclampsia are at the highest risk [53]. Furthermore, the association of PE with future cardiovascular mortality and morbidity is well established and has been replicated in diverse populations across multiple studies [54].

In 2017, the Combined Multimarker Screening and Randomized Patient Treatment with Aspirin for Evidence-Based Preeclampsia Prevention (ASPRE) trial showed that treatment with aspirin at a dose of 150 mg per day starting from 11 to 14 weeks of gestation until term in at-risk women resulted in a 62% reduction in the incidence of preterm PE, compared with placebo [55]. Therefore, screening in the first trimester of pregnancy would identify at-risk women who may have the greatest benefit from preventative measures [56]. We identified 39 studies on risk prediction models for HDP. Of these, only four models included GH as an outcome of interest, while the majority were targeted at PE risk in the first trimester.

### 4.1. Clinical Risk Factors for PE

An extensive systematic review and meta-analysis of 92 studies, including over 25 million pregnancies, demonstrated that the most significant risk factors for PE were that of a history of PE (relative risk (RR) 8.4), followed by chronic hypertension (RR 5.1), pregestational diabetes (RR 3.7), pregestational BMI > 30 kg/m [2] (RR 2.8) and use of assisted reproductive technology (RR 1.8) [57]. Clinical practice guidelines by several professional organisations, such as The National Institute for Care and Health Excellence (NICE), American College of Obstetricians and Gynaecologists (ACOG), and the International Society for the Study of Hypertension in Pregnancy (ISSHP) have proposed screening for PE based on maternal risk factors. These guidelines agreed on clinical risk factors for women at high-risk of PE, such as a previous pregnancy with PE, multifoetal gestation, the presence of chronic hypertension, autoimmune conditions (such as antiphospholipid syndrome or systemic lupus erythematosus (SLE)), chronic kidney disease and type 1 or type 2 diabetes mellitus. Moderate risk factors for PE include nulliparity, advanced maternal age (>35 years), obesity (BMI ≥ 30 kg/m [2]), family history of PE and booking systolic BP ≥ 130 mmHg or diastolic BP ≥ 90 mmHg [58].

The ACOG and NICE approach essentially treats each risk factor as a separate screening test, with additive detection rate and screen-positive rate. Furthermore, these methods result in binary classification of risk, with consequent suboptimal PE detection rates. Using the NICE recommendations, only 41% and 34% of preterm and term preeclampsia were detected, respectively, with a 10% false-positive rate. Screening based on the ACOG guidelines achieved detection rates of 5% and 2% for preterm and term PE, respectively, with a 0.2% false-positive rate [58].

### 4.2. Biomarkers and Biophysical Factors in PE Development

Considerable efforts have been made to identify biomarkers that can enhance PE prediction in the first trimester of pregnancy. Biomarker values are transformed into multiples of the median (MoM) to account for the effects of gestational age and maternal characteristics, such as weight and racial origin, associated with individual biomarkers. The use of combination biomarkers appears to confer superior predictive performance over a single biomarker [58]. Currently, combined biomarkers of PE include cytokines, proteins, angiogenic and antiangiogenic factors that have a fundamental role in pathophysiology [59]. Angiogenic factors, including soluble fms-like tyrosine kinase 1 (sFlt-1) and placental growth factor (PIGF), are involved in the pathogenesis of placental dysfunction. PIGF is pro-angiogenic and abundantly expressed in the placenta, while sFlt-1 is anti-angiogenic and regulates angiogenic homeostasis during pregnancy. An imbalance between pro- and anti-angiogenic factors (i.e., increased sFlt-1/PIGF ratio) results in a net anti-angiogenic state and favours the development of placental dysfunction [60]. Altered levels of sFlt-1 and PIGF are detectable several weeks before onset of pregnancy complications, thus allowing identification of women at high risk of PE. A recent systematic review of PE prediction models showed that up to 73% of preeclampsia cases could potentially be detected using a model that included serum markers such as PIGF, sFlt- and the mean arterial pressure (MAP) at 35–37 weeks’ gestation, compared to 35% with a set of maternal characteristics assessed between 9- and 13-weeks’ gestation [61]. In another meta-analysis, the predictive performance of PIGF in asymptomatic women demonstrated high predictive odd ratios for PE, with moderate sensitivity and specificity. Additionally, the accuracy of PIGF was higher when performed after 14 weeks’ gestation for the prediction of early onset PE [62].

PAPP-A, a pregnancy-related protease involved in early placental development, has been found to be dysregulated in intrauterine growth restriction, PE, placental abruption, and premature birth. Dysregulation of this enzyme is linked to a variety of pregnancy-related complications associated with placental function. However, the addition of PAPP-A to prediction models which already included maternal characteristics, PIGF and/or mean arterial pressure, did not significantly improve model performance [63,64]. While PAPP-A is a nonspecific marker of PE, it may have diagnostic application in combination with other clinical measurements and biomarkers [59]. Neutrophil gelatinase-associated lipocalin (NGAL), a lipocalin-type glycoprotein involved in iron sequestration and associated with inflammation, neoplastic transformation and renal damage, appears to be proportional to the severity of PE in the late second trimester of pregnancy, with sensitivity and specificity of 75% and 94.5%, respectively [65]. Elevated NGAL levels in early pregnancy may point to links between impaired immune tolerance and PE. While novel, NGAL is not linked exclusively to placental maladaptation, and its role in PE is currently unclear.

The most common measures of haemodynamic variables employed in PE risk prediction models are MAP and uterine artery pulsatility index (UtA-PI). MAP is obtained from the average of four measurements from two BP readings, one from each arm, taken simultaneously in a seated position. UtA-PI is measured at the level of the internal cervical os using transabdominal ultrasound. Pulsed wave Doppler is performed and the UtA-PI and peak systolic velocity are measured when three similar consecutive waveforms are obtained. A study evaluating low-risk Latin-American women in the first trimester of pregnancy found that the detection rate for preterm preeclampsia was 62.1% (AUC 0.817) based on maternal characteristics, which increased to 72.4% (AUC 0.890) with the inclusion of MAP and uterine artery Doppler measurements [66]. Antwi and colleagues found that predictive ability for gestational hypertension in Ghanaian women improved with the incorporation PAPP-A and PIGF measurements between 8 to 13 weeks of gestation [67].

In a large multi-centre study across seven regions in Asia, Chaemsaithong and colleagues observed that the MoM of biophysical parameters and biomarkers (MAP, UtA-PI and PIGF) showed more separation in earlier than later gestations, suggesting that these biomarkers had better discriminatory power for predicting preterm, rather than term PE [68]. While the use of certain biomarkers has been shown to enhance model performance, it should also be acknowledged that these biomarkers are prone to considerable variability in measurements, which are largely dependent on ethnicity, protocol adherence and quality assessment [58].

### 4.3. Risk Prediction Models for HDP/PE

The majority of risk prediction models evaluated in this review have focused on prediction of PE alone, with only five studies including both gestational hypertension and PE. These models employed a combination of maternal characteristics and biomarkers for HDP/PE prediction, with moderate detection rates. The Foetal Medicine Foundation (FMF) first trimester prediction model consists of a combination of maternal factors (age, BMI, race, smoking during pregnancy, family history of PE, method of conception, parity, presence of chronic hypertension, type 1 or type 2 diabetes mellitus, SLE, anti-phospholipid syndrome), and biophysical measurements (MAP, UtA-PI, PIGF and PAPP-A) [69]. Unlike models developed from using logistic regression analyses, the FMF algorithm uses a Bayesian approach to combine a priori risk from maternal factors with biochemical and biophysical measurements to quantify patient-specific risk for PE. While other prediction models treat PE as a binary outcome, the competing risk model treats PE as an event in time, so that risk stratification can be performed at different stages of pregnancy [70,71,72]. The FMF model has undergone extensive internal and external validation in several geographically diverse cohorts [64,73,74,75,76], with detection rates of 90% and 75% for the prediction of early and preterm preeclampsia, respectively, with a 10% false-positive rate [76]. To date, the FMF model appears to be the most well-studied [76], and external validation studies have shown comparable predictive performance corresponding to the original studies [58].

A simple risk score derived from the maternal history component of the FMF algorithm appeared to provide clinically useful prediction for preterm preeclampsia in nulliparous women with a singleton pregnancy. Neither the FMF online risk calculator or mobile application offer offline functionality, which may limit implementation in low-resource or time-poor settings. This score was devised to circumvent biomarker analysis and/or access to an internet-connected computer, and considered maternal age, height and weight, ethnicity, method of conception, history of chronic hypertension, history of type 1 or 2 diabetes mellitus and family history of PE, with good model performance for PE prediction on external validation (AUC 0.792). While the inclusion of first trimester MAP, PAPP-A and PIGF did not improve predictive ability in this study, compared to larger-scale models, this was possibly due to relatively fewer case numbers, which was underpowered to detect small improvements in model performance [77].

### 4.4. Machine Learning Approaches

Machine learning algorithms based on these combination models have shown that the use of artificial intelligence (AI) can effectively predict PE [78,79]. Sufriyana et al. utilised big data from a nationwide health insurance dataset in Indonesia of over 23,000 pregnant women and applied a machine learning algorithm based on demographic variables in early pregnancy and diagnoses on previous visits. This model had robust performance on both internal and external validation and was conceivably applicable in low-resource settings [79]. Meanwhile, in a Korean study of over 11,000 women, Jhee et al. showed that the combined application of maternal factors and common antenatal laboratory data in various machine learning algorithms resulted in improved prediction performance of PE, compared to traditional statistical models [78]. In a smaller study of Chinese women, support vector machine algorithms based on a combination of epidemiological, haemodynamic and biochemical factors were found to improve model accuracy and discrimination of HDP [80].

### 4.5. Summary

Advances in PE screening have demonstrated that the use of combined maternal and biophysical factors in prediction models clearly supersede traditional checklist screening of maternal risk factors, where binary risk categorization does not accurately reflect an individual’s risk of PE. The use of biophysical parameters and biomarkers that can be measured in the first trimester of pregnancy (8–13 weeks of gestation), including MAP, UtA-PI, PAPP-A and PIGF, has been shown to enhance the prediction of PE, when used in combination with maternal characteristics. Since the publication of the ISSHP recommendations, at least three systematic reviews have been published to evaluate risk prediction models for hypertensive pregnancies [61,81,82]. The reviews similarly acknowledged that included prediction models incorporated a range of maternal characteristics, and biomarkers (Table 1) with variable performance, calling for further external validation and comparison of the most promising models to better inform implementation in clinical practice. The application of machine learning algorithms to existing prediction models may refine predictive performance and improve accessibility, even in resource-poor healthcare settings.

## 5. Discussion

Contemporary risk prediction models for GDM and HDP demonstrate moderately good performance, sensitivity and specificity, in general. Several models were developed from large-scale population studies and have subsequently undergone external validation across geographically and ethnically diverse cohorts, showing potential for widespread clinical application. For GDM prediction, the use of maternal characteristics alone, including maternal age, BMI, ethnicity, past history and/or family history of GDM, achieved moderate discriminative performance. This was marginally enhanced by the inclusion of biomarkers such as fasting plasma glucose, high molecular weight adiponectin, SHBG, HbA1c and PAPP-A levels. In the case of PE prediction, several studies have consistently proven that models using a combination of maternal characteristics (maternal age, BMI, ethnicity, smoking during pregnancy, family history of preeclampsia, method of conception, parity, pre-existing hypertension and diabetes mellitus, SLE, anti-phospholipid syndrome) and biophysical factors and/or biomarkers (MAP, UtA-PI, PIGF and PAPP-A), perform more robustly than those using maternal risk factors alone. The best performing models for GDM and HDP prediction from 2016 and 2018 onwards, respectively, are summarized in Table 2 and Table 3.

### 5.1. Implications for Clinical Practice and Future Research

Risk prediction in itself does not necessarily translate to improved clinical outcomes, unless timely and effective management is undertaken. Early interventions for GDM and HDP, such as lifestyle modifications, adhering to GWG targets and the use of pharmacological therapies, have important implications for both short-term pregnancy outcomes and long-term maternal cardiovascular health.

Maternal pre-existing obesity is a modifiable risk factor and represents an important opportunity for dietary and physical activity interventions at the preconception or early pregnancy stages. The risk of GDM and HDP may be partially mitigated through healthy lifestyle in pregnancy and limiting excessive GWG, based on a systematic review and meta-analysis of 117 randomised controlled trials with over 34,000 women [85]. Identifying those at high risk of metabolic complications enables targeted prevention before, during and beyond pregnancy.

Most PE prediction models have been developed specifically for first trimester screening, based on proven risk reduction with early treatment. In contrast, less than half of current GDM prediction models included women at 20 weeks’ gestation or under, representing a missed opportunity for earlier intervention, especially when effective lifestyle strategies exist. Current approaches for first trimester screening for aneuploidy and PE employ a combination of risk factors and biomarkers in a multivariate logistic regression model to determine individual risk. Certain biomarkers and biophysical measurements used to detect PE, such as PAPP-A, MAP and UtA-PI, have biological plausibility for GDM, and may be useful to incorporate in a consolidated, cost-effective approach to early screening for composite pregnancy complications [36].

Few models have been implemented into routine clinical practice, although the Teede model for GDM prediction has been integrated into national guidelines in the Netherlands [86], and the FMF model for PE prediction has been endorsed by the International Federation of Obstetrics and Gynaecology (FIGO) as a one-step screening procedure in the first trimester [87].

Research priorities addressing cardiometabolic risk factors in pregnancy and beyond have been established, as women with GDM and/or HDP have substantially increased risk of future CVD, particularly in low- and middle-income countries [88,89]. Although GDM and HDP are conditions that typically manifest in the later stages of pregnancy, the first trimester is an emerging and important screening period for the prediction and prevention of these adverse outcomes. There is both biological plausibility and pragmatic rationale for development of a risk prediction model for composite cardiometabolic outcomes in early pregnancy, given the availability of shared maternal clinical characteristics and biomarkers that are routinely obtained in the first trimester. Such a model could be built on based on existing validated and/or high-performing models and be adapted to different resource settings.

Prediction models show promise in delivering personalised risk-stratification and management, in an era where precision medicine is advancing as the standard of care. The use of AI techniques in prediction modelling can improve model accuracy and precision, while natural language processing of big data could help inform model interpretation and pathogenic implications [79]. Machine learning methods often classify patients into homogenous categories, leading to poor prediction for heterogeneous conditions with wide clinical spectrums, in the case of GDM and HDP. Subtyping patients into well-defined and clinically meaningful clusters could train models for specific patient subtypes. These considerations underscore the importance of further research into patient subtyping in conjunction with supervised machine learning methods [90]. The widespread applicability of these models may be also limited by resource availability, ease of clinician use and patient acceptability for interventions based on risk prediction, rather than diagnostic tests. Finally, the impact of model implementation or uptake in clinical practice has not been adequately evaluated. A recent study found that a GDM risk prediction tool was generally well received by healthcare professionals and patients, with high clinician adherence rates [28]. The application of the FMF screening program in a London tertiary hospital led to a significant reduction in the PE screen-positive rate and a concurrent in increase in targeted aspirin use in women classified as high risk, demonstrating that this is both feasible and effective in a public healthcare setting [91].

### 5.2. Conclusions

Pregnancy adverse outcomes are a harbinger of future CVD risk in women. Systematic screening, especially in the first trimester, provides a window for opportunistic interventions that can translate into maternal and foetal well-being, beyond the confines of pregnancy. Further research is needed to evaluate the impact of implementation of current validated models, and to build on current models with novel biochemical, biophysical and molecular markers to enhance predictive performance. Machine learning algorithms appear to be comparable to established logistic regression models, with the potential for improving predictive accuracy. Furthermore, consideration should be given to developing a composite risk prediction model for GDM and/or HDP in early pregnancy, given the similarities in pathophysiology and prevention approaches for both conditions.

## Figures and Tables

**Table 1:** Candidate predictors for GDM and HDP/PE.

	GDM	HDP/PE
Clinical risk factors	Maternal age Ethnicity BMI or weight/height Smoking, alcohol and/or drug use Medical history (Pre-existing HTN, PCOS) Parity/Gravidity Obstetric history (including prior GDM, SGA baby and/or macrosomia) Family history of T2DM Systolic BP	Maternal age Ethnicity BMI or weight/height Smoking, alcohol and/or drug use Medical history (pre-existing HTN, SLE, APS) Parity/Gravidity Obstetric history (including prior HDP or SGA) Current/prior GDM Family history of HDP Method of conception Education Social class/income MAP Systolic BP
Biomarkers	Fasting plasma glucose HbA1c Triglycerides Adiponectin SHBG PAPP-A bHCG uE3 INH Urinary albumin Leptin Lipocalin-2 PAI-2	Blood glucose Triglycerides, TC, HDL-C, LDL-C ADAM12 PAPP-A sFlt-1 PIGF bHCG AFP VEGF
Radiological characteristics	N/A	Ultrasound Placental volume UtA Doppler UtA-PI Foetal biometry

BMI, body mass index; HTN, hypertension; PCOS, polycystic ovary syndrome; GDM, gestational diabetes; SGA, small-for-gestational age; T2DM, type 2 diabetes mellitus; BP, blood pressure; SLE, systemic lupus erythematosus; APS, antiphospholipid syndrome; HDP, hypertensive disorders of pregnancy; MAP, mean arterial pressure; HbA1c, haemoglobin A1c; SHBG, sex hormone binding globulin; PAPP-A, pregnancy-associated plasma protein-A; bHCG, beta human chorionic gonadotrophin; uE3, estriol; INH, dimeric inhibin-A; PAI-2, plasminogen activator inhibitor-2; TC, total cholesterol; HDL-C, high density lipoprotein-cholesterol; LDL-C, low-density lipoprotein-cholesterol; ADAM12, disintegrin and metalloproteinase domain-containing protein 12; sFLt-1, Soluble fms-like tyrosine kinase-1; PIGF, placental growth factor; UtA, uterine artery; UtA-PI, uterine artery-pulsatility index.

**Table 2:** Model performance metrics for GDM prediction.

First Author, Year of Study	Country of Study	Model	AUC	Sensitivity (%)	Specificity (%)	PPV (%)	NPV (%)
Benhalima, 2020 [39]	Belgium	Model 1: clinical variables (cut-off ≥4%)	0.68 (0.64–0.72)	99.1 (96.9–99.9)	4.4 (3.4–5.5)	12.9 (11.4–14.6)	97.2 (90.3–99.7)
		Model 2: clinical + biochemical variables (cut-off ≥4%)	0.72 (0.66–0.78)	94.2 (90.4–96.9)	13.7 (12.1–15.5)	13.5 (11.8–15.3)	94.3 (90.5–97.0)
Donovan, 2019 [33]	USA	California cohort	0.732 (0.728–0.735)	70.8 (70.2,71.4)	63.9 (63.7,64.0)	11.6 (11.4–11.8)	97 (97–97.1)
		California cohort (Black)	0.719 (0.700, 0.738)	49.3 (45.7, 53.0)	80.2 (79.6, 80.8)	9.0 (8.1, 9.9)	97.6 (97.3, 97.8)
		California cohort (Hispanic)	0.739 (0.733, 0.745)	65.0 (64.0, 66.1)	70.6 (70.3, 70.8)	11.3 (11.0, 11.6)	97.2 (97.1, 97.3)
Gao, 2020 [46]	China	Model 1: First antenatal visit screening at suggested risk score cut-off of 2.80	0.710 (0.680–0.741)	82.1	44.8	11.2	96.7
	China	Model 2: Other risk factors during pregnancy at suggested risk score cut-off of 5.10	0.712 (0.682–0.743)	81.8	44.4	11.1	96.6
Snyder, 2020 [34]	USA	Model 1: maternal characteristics only at 6% predicted risk threshold	0.714 (0.703–0.724)	76.2	55.2	-	-
		Model 2: maternal characteristics + first trimester PAPP-A at 6% predicted risk threshold	0.718 (0.707–0.728)	75.7	55.5	-	-
		Model 3: maternal characteristics + PAPP-A, uE3, and INH	0.722 (0.712–0.733)	76.1	55	-	
Sweeting, 2018 [36]	Australia	Model 1: Clinical parameters + First trimester markers	0.90 (0.87–0.92)	-	-	-	-
		Model 2 Early GDM: clinical parameters + First trimester markers	0.96 (0.94–0.98)	-	-	-	-
Sweeting, 2019 [35]	Australia	Sweeting 2018 model + adipogenic and metabolic syndrome markers (early GDM)	0.93 (0.89–0.96)	-	-	-	-
		Sweeting 2018 model + adipogenic and metabolic syndrome markers (overall GDM)	0.91 (0.89–0.94)	-	-	-	-
Theriault, 2016 [40]	Canada	Model 1: GDM (biomarkers and clinical variables) at 10% false positive rate	0.791 (0.750–0.831)	50	-	20.6	97.1
Zhang, 2020 [83]	China	Nomogram of GDM risk first trimester	0.728 (0.683–0.772)	71.6	65.2	50.2	89.5

AUC, area under the curve, PPV, positive predictive value; NPV, negative predictive value; PAPP-A, pregnancy-associated plasma protein-A; uE3, estriol; INH, dimeric inhibin-A.

**Table 3:** Model performance metrics for HDP prediction.

First Author, Year of Study	Country of Study	Outcome	Model	AUC	Detection Rate/Sensitivity (%)	Specificity (%)	PPV (%)	NPV (%)
Guizani, 2018 [64]	Belgium	PE at < 37 weeks	FMF algorithm	0.932 (0.923–0.940)	80.6 (64.0–91.8)	-	8	0.2
		PE at ≥ 37 weeks	FMF algorithm	0.741 (0.726–0.756)	31.8 (18.6–47.6)	-	3.2	0.8
Chaemsai-thong, 2019 [68]	Hong Kong, Japan, China, Thailand, Taiwan, India, Singapore	Preterm PE (FMF previous risk) at 20% FPR	FMF algorithm	0.758 (0.749–0.766)	57.52	-	-	-
		Preterm PE (FMF triple test) at 20% FPR	FMF algorithm	0.857 (0.851–0.864)	75.8	-	-	-
		All PE (FMF previous risk) at 20% FPR	FMF algorithm	0.711 (0.703–0.720)	52.38	-	-	-
		All PE (FMF triple risks) at 20% FPR	FMF algorithm	0.769 (0.761–0.777)	65.57	-	-	-
Wright, 2019 [84]	England, Spain, Belgium, Italy, and Greece	Early PE at 10% FPR	FMF algorithm	0.95 (0.93, 0.97)	87 (80, 92)	-	-	-
		Early PE at 10% FPR (SQS)		0.97 (0.95, 0.99)	93 (76, 99)	-	-	-
		Early PE at 10% FPR (SPREE)		0.96 (0.93, 0.98)	90 (78, 96)	-	-	-
		Preterm PE at 10% FPR	FMF algorithm	0.91 (0.89, 0.93)	75 (70, 80)	-	-	-
		Preterm PE at 10% FPR (SQS)		0.93 (0.89, 0.96)	75 (62, 85)	-	-	-
		Preterm PE at 10% FPR (SPREE)		0.93 (0.92, 0.95)	83 (76, 89)	-	-	-
		All PE at 10% FPR	FMF algorithm	0.83 (0.81, 0.84)	52 (49, 55)	-	-	-
		All PE at 10% FPR (SQS)		0.82 (0.80, 0.85)	49 (43, 56)	-	-	-
		All PE at 10% FPR (SPREE)		0.85 (0.83, 0.87)	53 (49, 58)	-	-	-
Sovio, 2019 [77]	UK	Preterm PE (NICE guidelines)	Logistic regression		53.6 (34.3–71.8)	89.4 (88.4–90.3)	3.3 (2.0–5.4)	99.7 (99.4–99.8)
		Preterm (PE) Derived Risk score from PGAPE	Logistic regression	0.846 (0.787–0.906)	57.1 (37.5–74.8)	91.2 (90.3–92.0)	4.2 (2.6–6.7)	99.7 (99.4–99.8)
		Preterm (PE) original ASPRE algorithm/ PGAPE	Logistic regression	0.854 (0.795–0.914)	60.7 (40.8–77.6)	90.4 (89.5–91.3)	4.1 (2.6–6.5)	99.7 (99.5–99.8)

AUC, area under the curve, PPV, positive predictive value; NPV, negative predictive value; FPR, false positive rate; FMF, Foetal Medicine Foundation; NICE, National Institute for Health and Care Excellence; SPREE, Screening programme for pre-eclampsia; SQS, Screening Quality Study; PGAPE, predicted gestational age at pre-eclampsia; ASPRE, Combined Multimarker Screening and Randomized Patient Treatment with Aspirin for Evidence-Based Preeclampsia Prevention.

## Data Availability

Not applicable.
